# Establishment of an immortalized human subglottic epithelial cell line

**DOI:** 10.1002/lary.27761

**Published:** 2019-01-08

**Authors:** Jason Powell, Bernard Verdon, Janet A. Wilson, A. John Simpson, Jeffery Pearson, Chris Ward

**Affiliations:** ^1^ Department of Otolaryngology–Head and Neck Surgery Freeman Hospital, Newcastle upon Tyne Hospitals NHS Foundation Trust Newcastle upon Tyne United Kingdom; ^2^ Institute of Cellular Medicine Newcastle upon Tyne United Kingdom; ^3^ Institute for Cell and Molecular Biosciences Newcastle upon Tyne United Kingdom; ^4^ Institute of Health and Society Newcastle University Newcastle upon Tyne United Kingdom

**Keywords:** Larynx, trachea, cell line, laryngostenosis

## Abstract

**Objective:**

Translational research into subglottic disease is restricted by the availability of primary human tissue originating from this subsite. Primary epithelial cells are also limited by their inability to survive beyond several divisions in culture outside of the body. Specific subglottic cell lines, useful for in vitro studies, have not yet been described. We therefore demonstrate what we believe to be the first immortalized subglottic epithelial cell line.

**Methods:**

Subglottic tissue was derived from a single adult patient's neoplasia‐free human subglottic brushing specimen. Cells were immortalized using a lentiviral vector expressing simian virus 40 T antigen. Karyotyping was performed on the transformed cells using single nucleotide polymorphism array comparative genomic hybridization. Transformed cells were phenotypically characterized by light microscopy, immunohistochemistry, and electrophysiology studies.

**Results:**

The immortalized subglottic cell line (SG01) was able to divide successfully beyond 20 passages. Karyotyping demonstrated no significant genomic imbalance after immortalization. The cells demonstrated normal epithelial morphology and cytokeratin expression throughout. SG01 cells were also successfully cultured at air–liquid interface (ALI). At ALI cells demonstrated cilia, mucus production, and relevant ion channel expression.

**Conclusion:**

The novel SG01 subglottic epithelial cell line has been established. This cell line provides a unique resource for researchers to investigate subglottic diseases, such as subglottic stenosis.

**Level of Evidence:**

NA. *Laryngoscope*, 129:2640–2645, 2019

## INTRODUCTION

The subglottis is a crucial site for airway infection, inflammation, and malignancy.^1^, ^2^, ^3^ Subglottic stenosis is one of the most common pathologies in this region. Found in both children and adults, it can result in potentially life‐threatening airway obstruction.^4^ Approximately 90% of all cases of acquired subglottic stenosis result from endotracheal intubation, with a reported rate of stenosis following intubation ranging from 0.9% to 8.3%.^5^ The subglottis is also a potential site of laryngeal cancer.^6^ Although rare, subglottic malignancy is nonetheless associated with late presentation and a poor prognosis.^6^ The subglottis also has a crucial relationship with the lower airway in terms of infection.^7^ This importance is epitomized in intubated and mechanically ventilated patients at high risk of ventilator‐associated pneumonia for whom removal of infected secretions from the subglottis can reduce the incidence of pneumonia.^8^, ^9^

Translational research relies on human samples to perform in vitro experimentation, such as eliciting disease pathophysiology or bench‐testing new potential therapeutic agents.^10^, ^11^, ^12^ This research is hampered by the availability of primary human tissue originating from this difficult to sample site.^10^, ^11^, ^12^ Previous researchers have used animal models or human tissue from other airway locations, such as the nose or lung; however, this is unlikely to fully recapitulate the subglottic airway in humans, which is a distinct respiratory niche.^13^, ^14^ We have previously reported the first description of primary human subglottic epithelial cell culture.^7^ Although establishing techniques for the sampling and culture of these primary cells is important, not all researchers will have access to these human samples. In addition, primary epithelial cells obtained from patients are limited by their inability to survive beyond several divisions in culture outside of the body.^15^ Specific subglottic cell lines, useful for in vitro studies, have not yet been described. We therefore demonstrate what we believe to be the first immortalized human subglottic epithelial cell line.

## MATERIALS AND METHODS

### *Collection of Subglottic Samples*

The appropriate ethical committees and hospital institutions granted approval for the study. Participants were recruited from otolaryngology–head and neck surgery waiting lists (Freeman Hospital, Newcastle upon Tyne, UK) prior to planned general anesthetic microlaryngoscopy. Exclusion criteria included patients with current or previous head and neck malignancy or with structural subglottic pathology. Subglottic mucosal brushings were obtained under direct microscopic vision in the operating room via a sheathed cytology brush (BC‐202D‐5010, Olympus, Japan), as previously described.^7^ The cytology brush was placed in a sterile container containing tissue culture medium (Roswell Park Memorial Institute‐1640, Sigma, Gillingham, UK) for immediate transport to the laboratory on ice.

### *Tissue Culture*

Epithelial cell culture and passage were performed using methods previously described.^7^, ^16^ In brief, samples were separated from the brush head, pelleted, and then cultured on collagen‐coated flasks in bronchial epithelial growth medium (BEGM) (CC‐4175, Lonza, Basel, Switzerland), supplemented with penicillin/streptomycin 100 U/mL (Sigma, Gillingham, UK), incubated at 37°C in a 5% CO_2_ incubator. Medium was changed every 2 to 3 days. Passage was performed when cells reached 70% to 80% confluence. The cells were removed from the culture surface with trypsin 0.05% (Sigma, Gillingham, UK), neutralized with 10% Fetal Bovine Serum (Sigma, Gillingham, UK), centrifuged, and then placed in a new growth container. Cells were also cultured at air–liquid interface (ALI), for which cells are exposed to air apically and medium basally. In these conditions, respiratory epithelial cells typically differentiate into a pseudostratified respiratory epithelium with ciliated and mucus‐producing cells.^17^ To achieve this, cells were passaged onto transwell inserts (0.4‐μm pore size, Corning, Corning, NY) and cultured with BEGM medium supplemented with SingleQuots (Lonza, Switzerland) and antibiotics.^16^, ^18^

### *Cell Immortalization*

Primary subglottic epithelial cells at passage 1 were transfected with a simian virus 40 (SV40) cell immortalization system, utilizing a recombinant lentiviral vector (106 IU/mL) (Applied Biological Materials, Inc., Vancouver, Canada), supplemented with 10 μg/mL Polybrene (Sigma). After transfection, cells were cultured as previously described. As per the manufacturer's instructions, the clonal population was selected by the ability of only the immortalized cells to survive senescence, which normally occurred in primary subglottic epithelial cells between three to five passages. Immortalization was confirmed by continuous culture past 20 passages. Epithelial morphology was confirmed on light microscopy. Comparison of immortalized cells was made with nonimmortalized primary subglottic epithelial cells, which have been extensively characterized by Powell et al.^7^

### *Karyotyping*

Single nucleotide polymorphism (SNP) array comparative genomic hybridization (CGH) was performed by the Northern Genetics Service, Newcastle upon Tyne, UK. DNA was extracted from two confluent culture flasks containing immortalized cells (passage 16) using the EZ1 DNA Tissue Kit (Qiagen, Hilden, Germany) on the EZ1 Advanced XL Robot (Qiagen, Germany). The DNA was quantified using standard procedures. SNP array CGH was carried out using whole genome Infinium CytoSNP‐850 K v1.1 BeadChip Array (Illumina, San Diego, CA). Analysis was performed on BlueFuse Multi v4.4 using Genome Build GRCh37 (Illumna, San Diego, CA). The backbone average resolution was approximately 50 kb.

### *Immunohistochemistry*

Cytokeratin staining was performed to confirm the epithelial phenotype. Cultures were fixed and permeabilized with methanol, then stained with monoclonal anti‐cytokeratin (pan‐reactive) antibody (raised in mouse), conjugated with Alexa Fluor 647 (Biolegend, San Diego, CA), counterstained with 4′,6‐diamidino‐2‐phenylindole dihydrochloride (DAPI) nuclear stain. Imaging was performed with a confocal fluorescent camera (Axio Imager II, Zeiss, Oberkochen, Germany).

### *Electrophysiological Studies*

Ussing chamber studies were used to characterize the electrophysiological properties of epithelial cells, such as relevant ion channel expression and transepithelial resistance. Experiments were performed using a static chamber system (World Precision Instruments, Sarasota FL).^19^ Apical and basolateral compartments were electrically isolated and separated by the polarized epithelial layer. A symmetrical Krebs solution was added to both compartments to eliminate potentially influential osmotic and chemical effects. Calomel voltage‐sensing electrodes were placed on each side of the membrane. Transepithelial potential difference was clamped to 0 mV by current injection with silver–silver chloride electrodes to eliminate the voltage gradient. 3 M potassium chloride salt bridges containing 3% agar were used to connect chambers to the electrodes. The chamber was maintained at 37°C and continuously gassed with 5% CO_2_/95% O_2_, pH 7.4. A 1 second 5 mV pulse was applied at 30‐second intervals to monitor resistance changes, calculated by applying Ohm's law. After 20 minutes of stabilization, relevant ion channel inhibitors and activators were added, and the resultant short‐circuit current (I_sc_) responses were recorded. Apical amiloride (Sigma) was added to inhibit epithelial sodium channels (ENaC). Apical forskolin (Sigma) was added to activate cystic fibrosis transmembrane conductance regulator (CFTR)‐mediated chloride transport through adenylate cyclase stimulation and intracellular cAMP increase. CFTR was inhibited by apical addition of CFTRinh172 (Sigma). The resultant analogue signal was digitized with a PowerLab 200 interface (AD instruments, Oxford, UK) and recorded by a computer with Scope 3.8.8 software (AD Instruments).

## RESULTS

Primary subglottic epithelial cell cultures were established from a neoplasia‐free human subglottic brushing specimen. The sample was obtained from a 65‐year‐old Caucasian male. The donor was a nonsmoker. The indication for microlaryngoscopy was persistent throat symptoms. No abnormality was identified on examination in the operating room. These subglottic primary cells were successfully immortalized to form SG01 cells. A second sample (from a separate patient) was also successfully immortalized to confirm the technique was reproducible.

**Figure 1:**
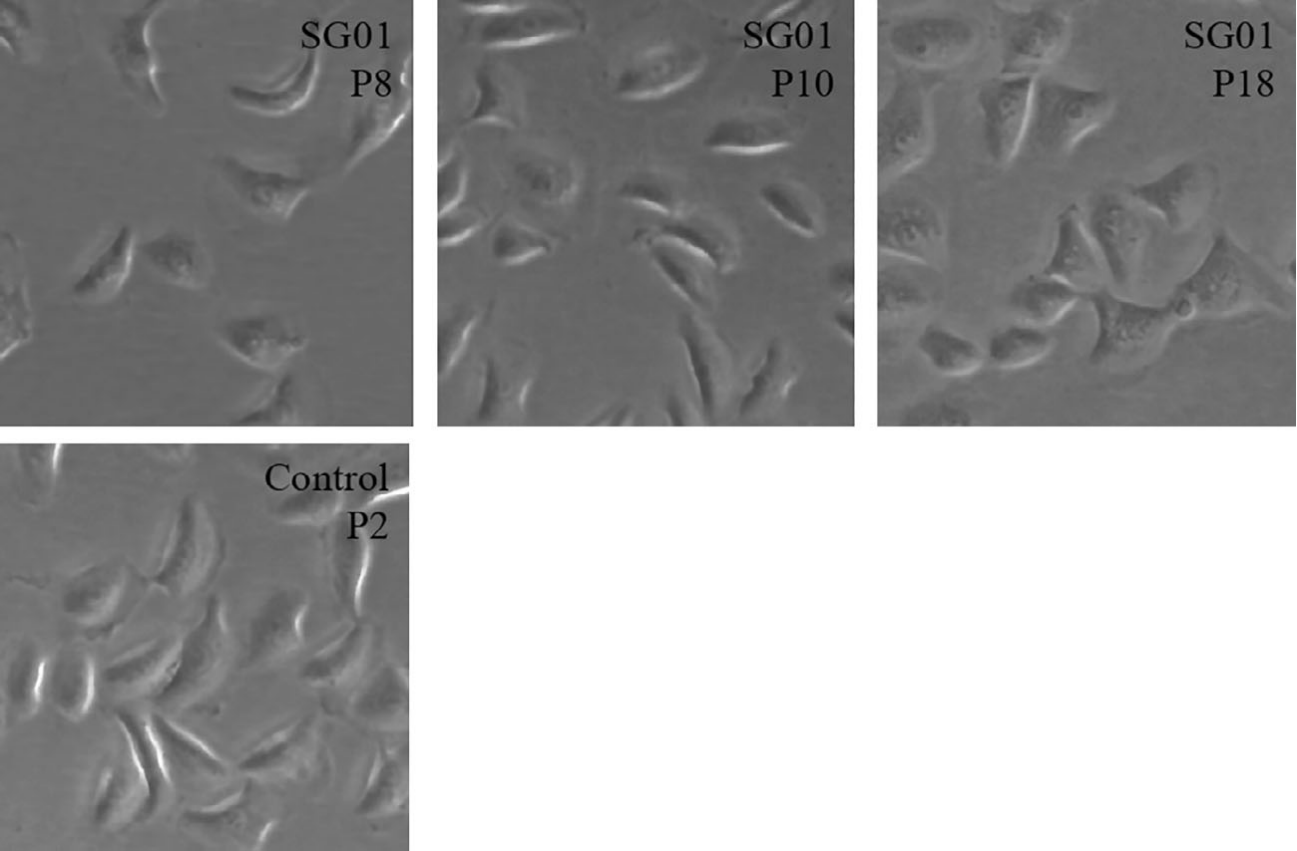
Brightfield light micrograph of immortalized (SG01) and nonimmortalized (control) subglottic epithelial cells cultured under submerged conditions (×20 magnification). Submerged cultures of SG01 cells demonstrate typical cobblestone appearance of respiratory epithelial cells. P = passage; SG01 = subglottic cell line.

**Figure 2:**
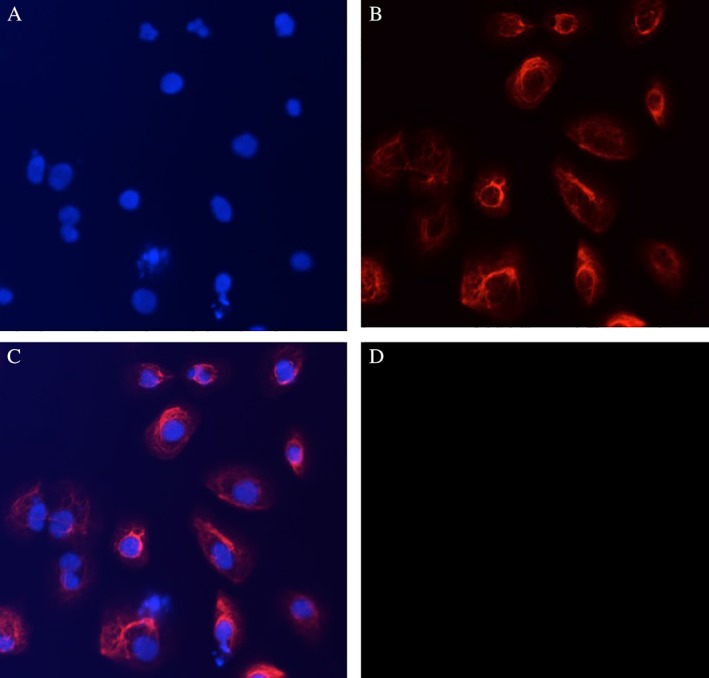
Pan‐cytokeratin staining of immortalized subglottic epithelial cells. Immortalized SG01 cells at passage 15 were fixed and stained with anti‐cytokeratin (pan‐reactive) antibody, then mounted in medium containing DAPI. A confocal fluorescent camera (Zeiss Axio Imager II, Germany) was used to image the cells. (A) DAPI nuclei stain (blue); (B) anti‐cytokeratin (pan‐reactive) antibody, conjugated with Alexa Fluor 647 (Biolegend, San Diego, CA) (red); (C) combined image of DAPI and cytokeratin; (D) background control to which the other images were set.DAPI = 4′,6‐diamidino‐2‐phenylindole dihydrochloride; SG01 = subglottic cell line. [Color figure can be viewed in the online issue, which is available at http://www.laryngoscope.com.]

**Figure 3:**
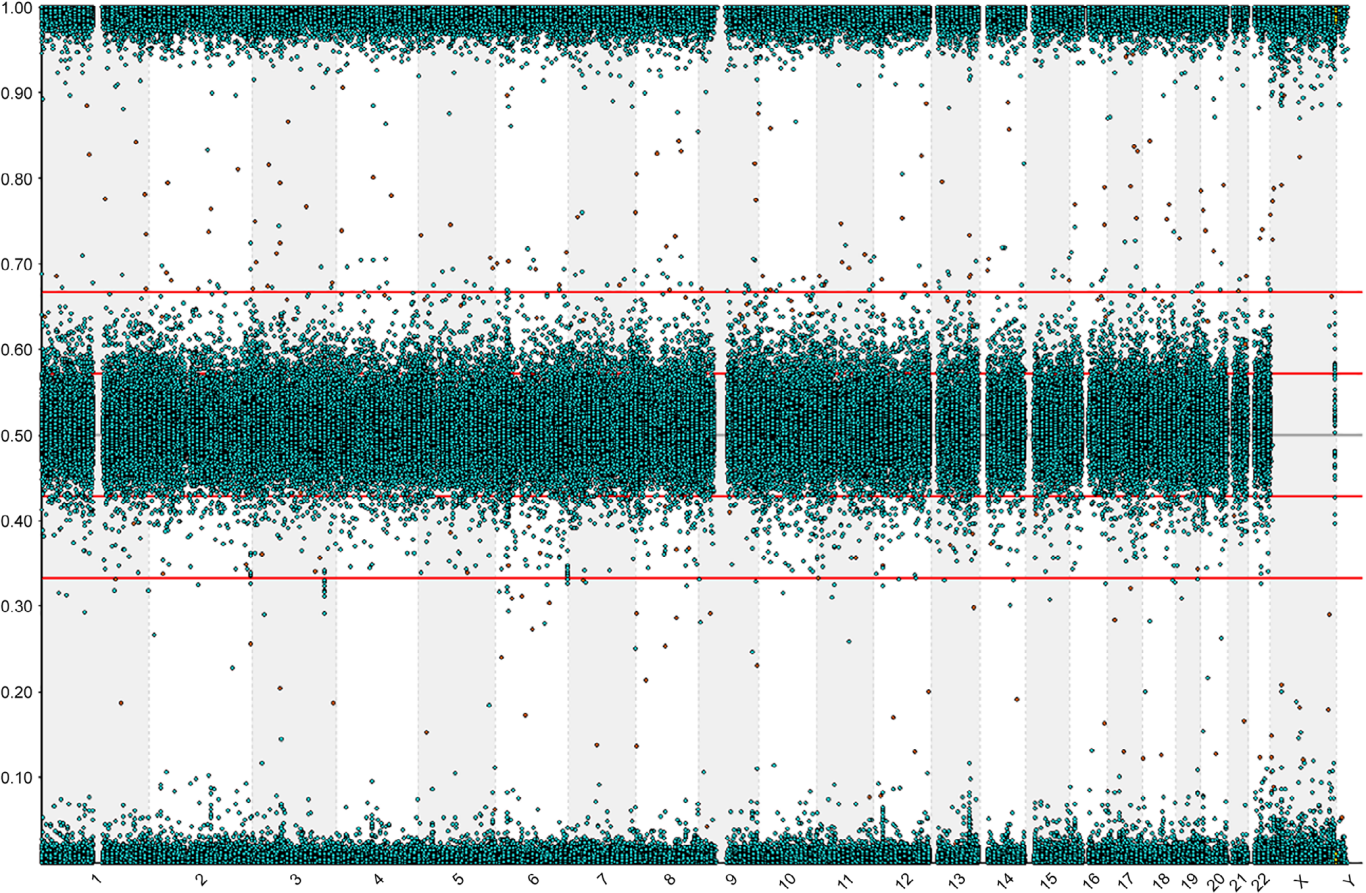
B‐allele chart demonstrating a male genotype with no significant genomic imbalance. Single nucleotide polymorphism array comparative genomic hybridization karyotyping was performed on DNA extracted from immortalized subglottic cell line cells at passage 16. B‐allele chart demonstrates chromosomal position (*x*‐axis) and b‐allele frequency (*y*‐axis), showing no significant genomic imbalance. [Color figure can be viewed in the online issue, which is available at http://www.laryngoscope.com.]

The immortalized subglottic epithelial cells (SG01 cells) display normal epithelial cell morphology on light microscopy (Fig. 1) and expression of cytokeratin, an epithelial cell marker (Fig. 2). SNP array CGH karyotyping demonstrated a male genotype with no significant genomic imbalance, no evidence of mosaicism, no loss of heterozygosity, and no uniparental disomy (Fig. 3). Cells have been expanded and cultured successfully beyond 20 passages.

**Figure 4:**
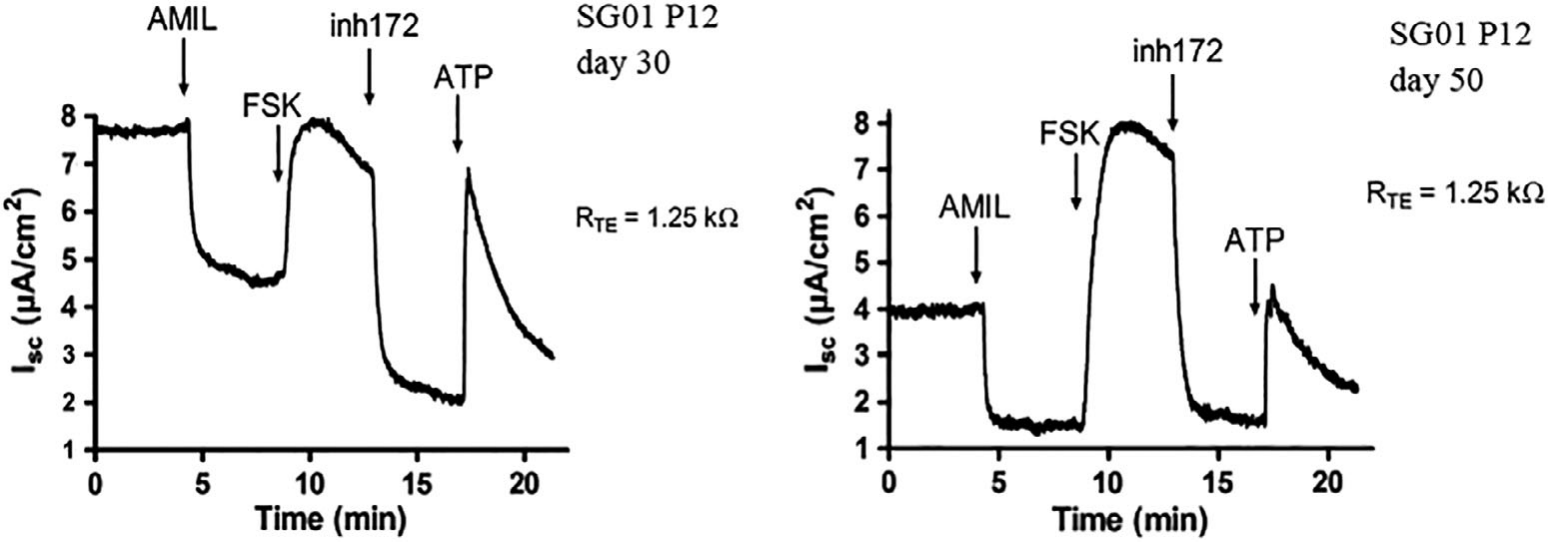
Ussing chamber experiments were performed on immortalized subglottic epithelial cells at passage 12 (day 30 and 50) at air–liquid interface culture. The Isc values reflect anion (Cl^−^) secretion and/or cation absorption (Na^+^). Transepithelial potential difference was measured (R_TE_). Immortalized cells demonstrated relevant ion channels expected of respiratory epithelial cells. AMIL was added to inhibit ENaC. Apical FSK was added to activate CFTR‐mediated chloride transport. CFTR was inhibited by apical addition of CFTRinh172. ATP was used to activate calcium‐activated chloride channels. AMIL = apical amiloride; ATP = adenosine triphosphate; CFTR = cystic fibrosis transmembrane conductance regulator; ENaC = epithelial sodium channels; FSK = forskolin; lsc = short‐circuit current; RTE = transepithelial tissue resistance; SG01 = subglottic cell line.

SG01 were also successfully lifted onto an ALI culture system. The cells had the apical fluid removed and differentiated by day 25. Brightfield light microscopy demonstrated, on repeated occasions, characteristic epithelial morphology and growth, including mucus production and cilia. Tight epithelial junctions were confirmed by resistance measurements on Ussing chamber experiments (Fig. 4). Relevant ion channel expression was also confirmed on Ussing chamber experiments, including ENaC and CFTR (Fig. 4).

## DISCUSSION

We describe, to our knowledge, the first immortalised human subglottic epithelial cell line, SG01. This model provides a unique resource for researchers to study subglottic diseases and potentially test therapeutic agents with a site‐specific in vitro model. We have confirmed that the SG01 cell line is highly representative of both primary in vitro cultures and the subglottic environment in vivo.

Valid experimental models are required to further elucidate the pathogenesis of subglottic diseases, such as subglottic stenosis and malignancy, and to develop therapeutic agents prior to human trials. The importance of cell culture models—in particular the immortalized epithelial cell models—in drug discovery and epithelial biology (including cancer biology) over the past half‐century cannot be overstated.^10^, ^11^, ^12^ The significant limitations of animal models in translational research have been extensively discussed elsewhere.^20^, ^21^, ^22^, ^23^ Translational researchers are increasingly reliant on appropriate in vitro models as an alternative to animal testing.^10^, ^11^ Primary cells, although superior to immortalized cell lines in terms of in vitro use, have significant limitations. The culture of primary cells is more invasive for patients, labor‐intensive for investigators, and expensive.^10^, ^11^ Primary cells are also limited by their finite lifespan outside of the body.^15^ Immortalized cell lines originate from one patient sample and are therefore more homogeneous. This removes interpatient sample variability between tests, making immortalized cells much more useful for the screening of large numbers of new drug candidates at low cost with high reliability and within a short time span.^10^, ^11^, ^12^

Human primary epithelial cell cultures and cell lines have previously been established from other airway locations, including the posterior commissure, trachea, and small airways of the lung.^16^, ^24^, ^25^, ^26^ These are, however, unlikely to reflect the subglottic region. The subglottis is an anatomically distinct region of the airway, differentiated from the trachea due to its circumferential binding to the cricoid cartilage, giving it unique physical properties.^13^, ^14^ These distinctive properties include a large number of seromucous glands present in the submucosa and a dense subepithelial capillary plexus with numerous anastomoses.^14^

Several methods exist for immortalizing mammalian cells in culture. Cells were immortalized using a lentiviral vector expressing SV40 T antigen. This mechanism of immortalization is well documented as having a stable genetic background. SV40 T antigen has been shown to be one of the most reliable agents for the immortalization of cells in culture, and the mechanism has been well documented.^27^, ^28^, ^29^, ^30^, ^31^ Multiple other human epithelial subsites have been immortalized utilizing SV40, including esophageal, corneal, ovarian, and urinary tract.^29^, ^32^, ^33^, ^34^ The technique has also been applied to nonepithelial cells, such as neural, epidermal, and endothelial cells, in humans and other mammals.^35^, ^36^, ^37^

A potential limitation of this model is that it utilized subglottic cells sampled from a patient undergoing microlaryngoscopy for the diagnosis of persistent upper airway symptoms. The examination was normal, and no subglottic pathology was encountered. However, these symptoms could be due to laryngopharyngeal reflux, which has been shown to alter laryngeal cell phenotype.^38^, ^39^, ^40^ The samples had to be obtained during general anesthesia due to the difficulty in accessing the subglottis; therefore, it was not possible to access subglottic samples from completely asymptomatic patients (who would not be undergoing this procedure). Another limitation of this study is the age of the patients sampled. Although subglottic malignancy is almost entirely found in adults, subglottic stenosis is more commonly found in children. To our knowledge, there is no specific literature comparing the pediatric and adult subglottis at an epithelial level. There is, however, the potential for difference. Establishment of a pediatric version of this cell line would be an area for further research.

## CONCLUSION

The novel SG01 subglottic epithelial cell line has been established. This cell line provides a unique resource for researchers to investigate subglottic diseases, such as subglottic stenosis. SG01 cells divide successfully beyond 20 passages; can differentiate at ALI culture; and demonstrate normal epithelial morphology, cytokeratin expression, and relevant ion channel expression.

## Author contributions

Jason Powell: acquisition of data, analysis and interpretation of data, drafting of the manuscript; Bernard Verdon: acquisition of data, analysis and interpretation of data, drafting of the manuscript; Janet Wilson: study supervision, study concept and design, drafting of the manuscript; John Simpson: study supervision, study concept and design, drafting of the manuscript; Jeffery Pearson: study supervision, study concept and design, drafting of the manuscript; Chris Ward: study supervision, study concept and design, drafting of the manuscript.
